# The Works of the Late Joseph Else, F. R. S. Surgeon to St. Thomas's Hospital, and Member of the Royal Academy of Surgery at Paris, Containing a Treatise on the Hydrocele, and Other Papers on Different Subjects in Surgery. To Which Is Added an Appendix, Containing Some Cases of the Hydrocele, with a Comparison of the Different Methods of Treating It by Caustic and Seton

**Published:** 1782

**Authors:** 


VII. *The Works of the late* Jofeph Elfe, F. R. S. *Surgeon to St. Thomas's Hofpital, and Member of the Royal Academy of Surgery at Paris, containing a treatife on the Hydrocele, and other papers on different fubjects in furgery. To which is added an appendix, containing fome cafes of the Hydrocele, with a comparifon of the different methods of treating it by cauftic and feton. By* George Vaux, *Surgeon.* 8vo. Johnfon, *London,* 1782. 144 pages.

BESIDES an effay on the hydrocele, which has defervedly paffed through feveral editions, Mr. Elfe was the author of feveral ingenious and ufeful papers publifhed in different volumes of the Medical Obfervations and Inquiries. All thefe pieces are brought together in in the prefent collection. To enter into any particular account of them in this work would be fuperfluous, as they have been fo long publifhed, and are in every medical library. We fhall therefore content ourfelves with noticing the remarks publifhed by the editor of the volume in his appendix to the treatife on the hydrocele.

Mr. Elfe's principal view was to recommend, for a radical cure of this difeafe, the operation by cauftic, as a remedy in point of eafe, expedition, fafety and efficacy, far fuperior to every other that has been propofed, and particularly to the operation by a feton.

On the other hand, Mr. Pott, in his treatife on the hydrocele, recommends, on fimilar grounds, as a radical cure for the difeafe, the operation by a feton, which he affirms to be preferable to every other, and efpecially to that by means of a cauftic.

The queftion relative to the preference which either of thefe methods claims over the other, can be determined only by repeated comparative trials of both, this is what the editor has done, and his decifion is clearly in favour of the cure by cauftic.

The operation by cauftic, he obferves, is lefs painful than that by a feton. He has feen many cafes cafes in which the operation has been performed by a feton; and in all of them the pain has been confiderable, and in fome extremely violent; whereas in the cafes in which he has feen the cauftic employed, he has never obferved the patient to fuffer any material pain.

With regard to the inconvenience and hazard, and the length of time taken up in the cure, fo far as Mr. Vaux's obfervation goes, the two methods will not admit of a comparifon. In the cafes where he has feen the feton employed, the operation has been generally followed by a fever and high inflammation. Thefe circumftances have rendered confinement abfolutely neceffary, and, by their violence, have fometimes endangered the patient's life. On the contrary, in thofe cafes where he has feen the cauftic ufed, as they were attended with very little pain, fo they were accompanied with no fever or inflammation worth notice, and of courfe little confinement has been neceffary.

In cafes where the feton had been employed, Mr. Vaux has obferved the cure to have failed altogether; or if the patient for the prefent appeared to be cured, the difeafe returned, fo that in either cafe another operation has become neceffary. On the other hand, in the cafes he has been been witneſs to, or has been informed of, where the operation has been originally performed exactly according to Mr. Elſe's directions, he has met with none in which this operation did not prove an effectual cure.

Mr. Vaux deſcribes his treatment of a double hydrocele. This caſe was advantageouſly calculated for the purpoſe of deciding the queſtion concerning the comparative merits of the two different operations; by affording an opportunity of employing in the ſame perſon, the cauſtic operation for the cure of the diſeaſe on one ſide of the ſcrotum, and the ſeton for the cure of that on the other.

A cauſtic was applied to one of the hydroceles, and after the inconveniences ariſing from this operation had abated, a ſeton was applied to the other. A radical cure was obtained by both methods; with this difference, that the inflammation and pain produced by the ſeton were extremely violent; whereas, the cauſtic hardly occaſioned any inconvenience to the patient.

A ſecond caſe of this kind, we are told, nearly agreed with the one juſt now related. In a third caſe of double hydrocele, the account of which is communicated by Mr. Ford, of the Weſtminſter Diſpenſary, one, and the largeſt of the hydroceles, was was cured by cauftic. To the other a feton was applied, which, after producing fevere and violent inflammation, feemed to have effected a complete cure, but at the end of eight months the diforder returned again on this fide, and recourfe was had to the cauftic. But this likewife proved ineffectual, and the complaint was at length radically cured by an incifion of the tunica vaginalis, through the whole length of the tumour. The failure of the cauftic operation in this inftance is attributed by the editor to the adhefions of the tunica vaginalis, brought on by the feton. It is added likewife, by Mr. Ford, that on examining the ftate of the tefticle he found a fmall hydatid on the epididymis, and neither the feton nor the cauftic have been recommended as a certain cure for the hydrocele when occafioned by hydatids.

Mr. Vaux is of opinion, that no fubftantial advantage is derived from the practice of mixing opium with the cauftic; but that, on the contrary, it tends to weaken the effect of the remedy. He obferves, that cauftic applied on found fkin gives little uneafinefs, although it never fails to excite great pain when it touches parts that are inflamed.

In cafes of hydrocele where the tumour is large, and the tunic much diftempered, Mr. Vaux advifes a larger cauftic to be applied than is generally ufed; and where the tumour is of uncommon magnitude, he thinks it would not be improper to apply the cauftic on two parts, fufficiently remote from each other, fo as to affect the whole of the fac.

Mr. Vaux concludes his obfervations with an account of two cafes, attended with fome uncommon circumftances.—In one of thefe, the tunica vaginalis, after the water had been drawn off by a trocar, became diftended with blood. In the other, the tunic being greatly difeafed, the puncture with the trocar brought on inflammation and abfcefs. The tunic floughed away through the wound, and the patient got perfectly well.

